# Future changes in the climatology of the Great Plains low-level jet derived from fine resolution multi-model simulations

**DOI:** 10.1038/s41598-017-05135-0

**Published:** 2017-07-10

**Authors:** Ying Tang, Julie Winkler, Shiyuan Zhong, Xindi Bian, Dana Doubler, Lejiang Yu, Claudia Walters

**Affiliations:** 1 Department of Geography, Environment and Spatial Sciences, Michigan State University, East Lansing, Michigan 48824 USA; 2 Northern Research Station, USDA Forest Service, Lansing, Michigan 48910 USA; 3 Department of Social Science, University of Michigan-Dearborn, Dearborn, Michigan 48128 USA

## Abstract

The southerly Great Plains low-level jet (GPLLJ) is one of the most significant circulation features of the central U.S. linking large-scale atmospheric circulation with the regional climate. GPLLJs transport heat and moisture, contribute to thunderstorm and severe weather formation, provide a corridor for the springtime migration of birds and insects, enhance wind energy availability, and disperse air pollution. We assess future changes in GPLLJ frequency using an eight member ensemble of dynamically-downscaled climate simulations for the mid-21st century. Nocturnal GPLLJ frequency is projected to increase in the southern plains in spring and in the central plains in summer, whereas current climatological patterns persist into the future for daytime and cool season GPLLJs. The relationship between future GPLLJ frequency and the extent and strength of anticyclonic airflow over eastern North America varies with season. Most simulations project a westward shift of anticyclonic airflow in summer, but uncertainty is larger for spring with only half of the simulations suggesting a westward expansion. The choice of regional climate model and the driving lateral boundary conditions have a large influence on the projected future changes in GPLLJ frequency and highlight the importance of multi-model ensembles to estimate the uncertainty surrounding the future GPLLJ climatology.

## Introduction

The Great Plains low-level jet (GPLLJ), a fast-moving southerly airstream in the lower troposphere, is one of the most important atmospheric circulation features influencing the central U.S. Roughly one-third of the moisture entering this region is transported from the Gulf of Mexico by the GPLLJ^1^, and convergence downstream of the jet promotes the formation of thunderstorms and convective precipitation^2^. GPLLJs also influence the intensity and longevity of mesoscale convective complexes^3^, and diurnal variations of the GPLLJ help explain the summertime nocturnal precipitation maximum of the central plains^4^. An anomalously strong GPLLJ contributed to the central U.S. floods of 1993^5^, 2008^6^, and 2015^7^, and increased (decreased) precipitation in the northern (southern) plains observed during 1979–2012 was attributed to a northward expansion of the GPLLJ^8^. Moreover, regional tornado activity is correlated with fluctuations in the GPLLJ’s strength and position^9^.

In addition to its hydrological impacts, the GPLLJ provides tailwind assistance for northward springtime bird migration, but impedes southward migration in fall^10^. Furthermore, the GPLLJ facilitates the migration of insect pests, such as potato leafhopper^11^ and green peach aphid^12^, which are responsible for millions of dollars of agricultural crop lost annually. The GPLLJ also contributes to the wind energy resources of the Great Plains with wind power potential estimated at 25% higher when a GPLLJ is present^13^, although the wind shear below the jet also poses a hazard to the lifetime and performance of wind turbines^14^. GPLLJs are a source of wind shear near airports affecting aviation safety^15^, and the mechanical mixing of the GPLLJ moderates urban heat island intensity of metropolitan areas in the Great Plains^16^. GPLLJs also impact air quality by transporting marine air from the Gulf of Mexico into the southern plains and polluted continental air into the central and northern plains^17^. GPLLJs additionally transport smoke from wildfires and have been linked to blow-up fire behavior^18^.

Physical processes at a range of spatial and temporal scales contribute to GPLLJ formation. GPLLJs are most frequent at night during the warm season from approximately April to October^19–21^. At this time of year, synoptic-scale southerly airflow arises from the deflection by the Rocky Mountains of the anticyclonic circulation around the North Atlantic Subtropical High (NASH)^22, 23^ and/or the geopotential height gradient established between the subtropical high and low pressure over the Rocky Mountains^24^. Boundary-layer processes interact with the southerly airflow. These include diurnal fluctuations in surface buoyancy due to differential heating along the sloped topography of the Great Plains^25, 26^, and a decrease in turbulent diffusivity after sunset accompanied by the development of an inertial oscillation near the top of the nocturnal boundary layer^26, 27^. Although not as frequent, GPLLJs also occur at other times of the day and year^19–21^. These jets are primarily forced by synoptic-scale mechanisms, such as strong upper-level jet streaks^28^ and developing extratropical cyclones^29, 30^. Daytime and/or cool-season jets tend to occur at higher elevations (>1000 m AGL) compared to the average elevation (400–600 m AGL) of nocturnal warm-season jets^21^, reflective of the differing forcing mechanisms.

Future changes in the climatology of the GPLLJ due to anthropogenic climate change potentially have substantial environmental and societal implications, particularly for water resources, agriculture, biodiversity, renewable energy, aviation, and air quality. Thus, projections of the future GPLLJ climatology that span the central U.S. and consider sub-seasonal and sub-daily variations are needed for resource planning. An assessment of the uncertainty surrounding the projections also is needed to assist stakeholders with robust and flexible decision making^31, 32^. Future changes in the GPLLJ climatology remain poorly investigated, however. The few previous studies were mostly confined to GPLLJs in the southern plains, with the GPLLJ defined simply as the meridional average wind speed on a constant (850 hPa or 925 hPa) pressure level^24, 33, 34^. This simplified jet definition does not consider the presence of vertical wind shear or diurnal and seasonal variations in jet elevation. Moreover, previous analyses that employed atmosphere-ocean general circulation model (AOGCM) simulations^24^ were limited by the models’ coarse spatial resolution, whereas those that dynamically-downscaled AOGCM simulations using regional climate models (RCMs)^33, 34^ were confined to a single RCM, thus ignoring uncertainty introduced by differences among RCMs in simulating the processes influencing jet occurrence^35^.

## Data and Methods

We use an ensemble of dynamically-downscaled climate simulations available from the North American Regional Climate Change Assessment Program (NARCCAP)^36^ to assess future changes in the GPLLJ climatology and the associated uncertainty. Only those NARCCAP simulations with wind information at multiple vertical levels are included in the analysis, for a total of eight RCM_AOGCM combinations obtained from four RCMs (CRCM, WRFG, RCM3, and HRM3) with four AOGCMs (CCSM, CGCM3, GFDL, and HADCM3) used to drive the RCMs at the lateral boundaries of their regional domains (see Table S1 for a listing of simulations and explanation of abbreviated model names). Simulations are available for baseline (1970–2000) and mid-century (2040–2070) periods. The mid-century climate projections are forced by the SRES A2 greenhouse gas emissions scenario^37^.

The RCM_AOGCM simulations, archived at 3-hourly time steps, have a 50 km horizontal resolution and a vertical resolution in the lower troposphere of 25 hPa. Vertical wind profiles at each model gridpoint and archived time step were queried for a southerly (113°–247°) lower-troposphere wind maximum of ≥12 ms^−1^, with at least a 6 ms^−1^ decrease in speed to the next minimum above and below the level of maximum wind speed to ensure the presence of the classic “nose” in the wind profile. This definition follows that used in numerous previous climatological analyses of the GPLLJ^19–21, 38^. The findings below are presented as “deltas” (i.e., change factors) between the future and baseline periods.

Simulations for historical and baseline periods are able to reproduce the observed broad-scale spatial patterns and diurnal and seasonal variations of the GPLLJ. In an earlier analysis, we evaluated the simulations from the four RCMs when driven by National Centers for Environmental Prediction (NCEP) Reanalysis II^39^ fields for a historical period against rawinsonde observations at 0000 UTC and 1200 UTC, and found that, in spite of the coarser vertical resolution of the NARCCAP simulations, the models simulate the major features of the observed GPLLJ climatology^40^. Moreover, when averaged across all locations and RCM simulations, the RCMs underestimate jet frequency by only 0.6% for warm season (April –September) jets at 0000 UTC and by 1.4% for the more frequent 1200 UTC warm season jets. Deviations are somewhat larger for cool season (October-March) jets (−1.6% and −4.1% for 0000 UTC and 1200 UTC, respectively). On average, the RCMs underestimate mean jet speed by less than 1 ms^−1^, and mean jet elevation is underestimated by less than 10 m for jets at 1200 UTC but by >100 m for 0000 UTC jets. Deviations vary by model, although no RCM consistently outperforms the others. We further compare monthly GPLLJ frequencies at 3-hourly time steps obtained from the RCM_AOGCM simulations for the baseline period to long-term (1979–2009) climatological values calculated^21^ from the North American Regional Reanalysis (NARR)^41^ at 0600 UTC for April through September (Figure S1). NARR is used for the comparison because of its higher spatial and temporal resolution compared to rawinsonde observations, although its vertical resolution is coarser and NARR is known to underestimate observed GPLLJ frequency^38, 42^. Thus, the comparison focuses on the spatial and seasonal patterns of the GPLLJ which NARR replicates well^21^, rather than the magnitude of jet frequency. The RCM_AOGCM combinations simulate a nighttime maximum in jet frequency during the warm season and relatively larger jet frequencies between approximately 90°–105°W, similar to the NARR climatology. Simulated jet frequencies increase from spring to mid-summer and decrease thereafter, as seen in the NARR climatology. The RCM_AOGCM simulations also capture the shift in the location of maximum jet frequency from the southern plains in spring to the central plains in summer. However, the seasonality of jet frequencies in the NARCCAP simulations is stronger than that for NARR, especially for the WRFG_CCSM, WRFG_CGCM3, and RCM3_CGCM3 simulations.

## Results

### Projected future changes in GPLLJ frequency

**Figure 1:**
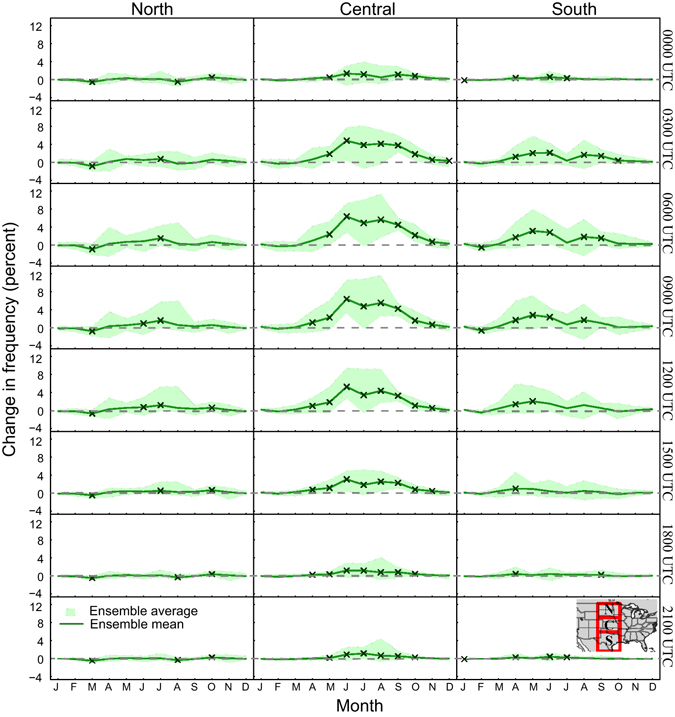
Difference of average GPLLJ frequency for 3 subregions over the Great Plains by month between the baseline (1970–2000) and mid-century (2040–2070) periods. Monthly frequencies are shown for each of the 3-hour time periods available for the NARCCAP simulations. The locations of the subregions are shown in the inset map. The solid green line is the multi-model mean difference between the two periods. The green shading depicts the uncertainty range obtained from the RCM_AOGCM simulations calculated as maximum difference minus minimum difference from the eight simulations. The “x” symbols indicate statistically significant differences (p = 0.10) in the multi-model means. This figure was created in R, version 3.3.1 (R Core Team. (2016). R: A language and environment for statistical computing. R Foundation for Statistical Computing, Vienna, Austria. https://www.R-project.org/).

In Fig. 1 we show the diurnal variations in the multi-model mean projected change in monthly GPLLJ frequency along with the associated uncertainty ranges, defined as the spread of the eight RCM_AOGCM simulations, for three spatial windows representing the northern, central, and southern plains (see inset map). For each spatial window, monthly jet frequencies were averaged across the RCM_AOGCM combinations by year for the baseline and mid-century periods, and the differences in the multi-model means between the two periods were tested for statistical significance using Student’s t-test with unequal variance^43^ (see Supplemental Information for more detail on the significance testing including results from alternative approaches). These results provide guidance for assessing the relative magnitude of the projected changes, but should be interpreted cautiously as the RCM_AOGCM simulations are not independent^44^. For the northern plains where GPLLJs are infrequent, the multi-model means of projected change in monthly jet frequency mostly are insignificant, although the model spread is larger during the nighttime and morning hours of the warm season. In contrast, the uncertainty range for the projected change in jet frequency for the central region is much wider from approximately May through October, especially for the nighttime and morning hours (0300-1500 UTC). Nevertheless, significant differences in nocturnal GPLLJ frequency are seen for most months, with the largest differences (>6%) in the regionally-averaged frequencies occurring in June-August. Moreover, the majority of the RCM_AOGCM simulations are in agreement with the sign of the projected change (Figure S2). Little change in jet frequency is expected during late morning and afternoon in the central plains, as suggested by the smaller projected changes for these time steps. A similar pattern is observed for the southern region with significant (insignificant) differences in the ensemble means for 0300-1200 UTC (1500-0000 UTC) and a wider uncertainty range during the warm season, although the differences are somewhat smaller than those for the central plains.

**Figure 2:**
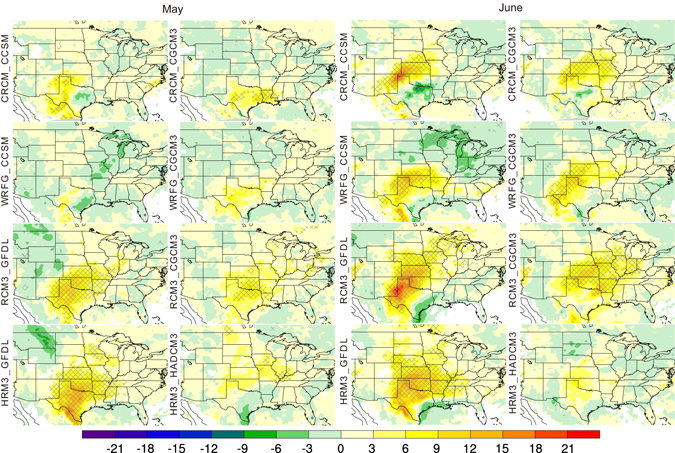
Projected change by mid-century in the frequency (in percent) of GPLLJs at 0600 UTC during May and June for each of the eight RCM_AOGCM simulations. Hatching indicates gridpoints with statistically significant differences (p = 0.10) between the mid-century and baseline periods. This figure was created using NCAR Command Language (NCL) Version 6.3.0 (The NCAR Command Language (Version 6.3.0) [Software]. (2016). Boulder, Colorado: UCAR/NCAR/CISL/TDD. http://dx.doi.org/10.5065/D6WD3XH5).

**Figure 3:**
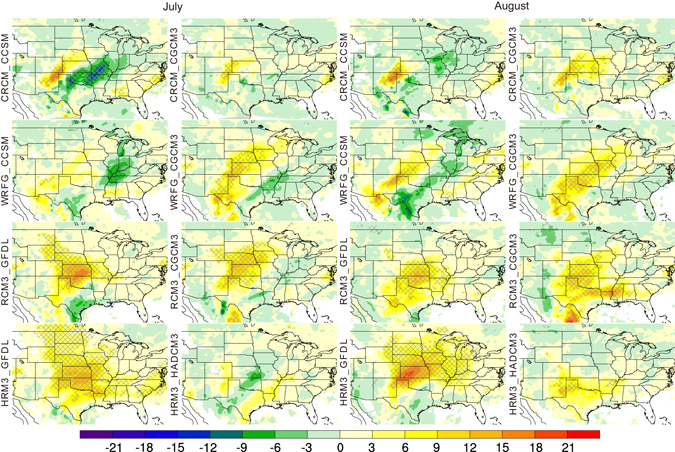
Projected change by mid-century in the frequency (in percent) of GPLLJs at 0600 UTC during July and August for each of the eight RCM_AOGCM simulations. Hatching indicates gridpoints with statistically significant differences (p = 0.10) between the mid-century and baseline periods. This figure was created using NCAR Command Language (NCL) Version 6.3.0 (The NCAR Command Language (Version 6.3.0) [Software]. (2016). Boulder, Colorado: UCAR/NCAR/CISL/TDD. http://dx.doi.org/10.5065/D6WD3XH5).

Differences in GPLLJ frequency between the mid-century and baseline conditions also were calculated at individual gridpoints for each of the RCM_AOGCM combinations and tested for statistical significance. Representative findings are presented for a nighttime time step (0600 UTC) for May-September. These plots highlight the changes during the warm season of the location of greatest projected changes and the differences among the RCM_AOGCM combinations. In May, spatially coherent areas of statistically significant increases in jet frequency are evident for only four simulations, with the largest changes (approaching 20% at some gridpoints) located in the southern plains (Fig. 2). By June, all but one of the simulations (the exception is HRM3_HADCM3) project coherent areas of significant increases in jet frequency with the largest changes located somewhat northward compared to May. Small, largely insignificant, decreases in jet frequency are found in the extreme southern plains. A striking feature of the plots for July and August (Fig. 3) is the southwest-northeast-oriented couplet of increased (~3–21%, depending on the simulation and month) jet frequency over the western plains and decreased jet frequency (as much as 15%) in the eastern plains and Midwest evident for the RCM simulations driven by CCSM (WRFG_CCSM, CRCM_CCSM) and, in July only, those driven by CGCM3 (WRFG_CGCM3, RCM3_CGCM3). Most of the remaining models project a broad area of increased jet frequencies (~3–24%) in the central plains, although the spatial pattern for HRM3_HADCM3 is in opposition with decreased jet frequencies over the central plains. The majority of the simulations project increased jet frequencies compared to the baseline period in the central plains during September, although the magnitude of the projected changes is generally smaller compared to previous months (Figure S3).

### Projected changes in synoptic-scale airflow

**Figure 4:**
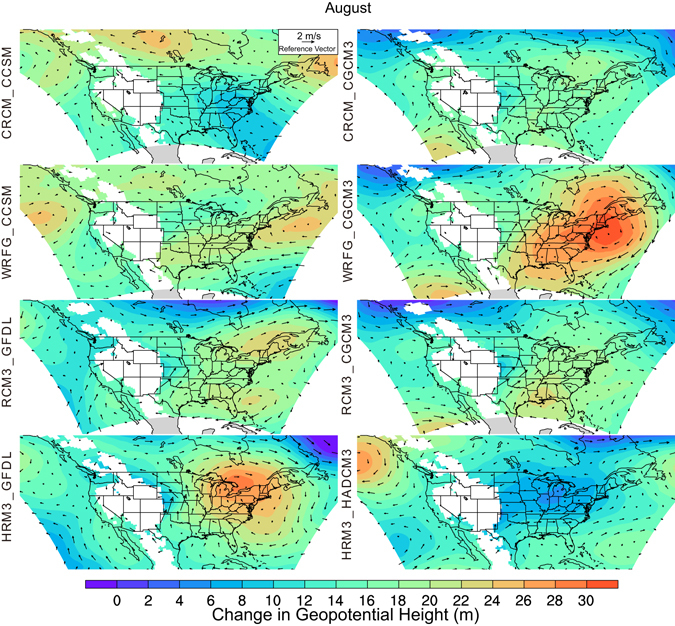
Differences in 850-hPa geopotential height (shading) and 850-hPa wind (vectors) between the mid-century and baseline periods at 0600 UTC for August for each of the eight RCM_AOGCM simulations. Gridpoints with elevations greater than 1400 m above sea level are masked in white. This figure was created using NCAR Command Language (NCL) Version 6.3.0 (The NCAR Command Language (Version 6.3.0) [Software]. (2016). Boulder, Colorado: UCAR/NCAR/CISL/TDD. http://dx.doi.org/10.5065/D6WD3XH5).

As mentioned above, GPLLJs during the warm season often form when boundary-layer processes act on synoptic-scale, anticyclonic airflow around the NASH and/or the geopotential height gradient established between the NASH and low pressure over the Rocky Mountains^22–24^. To assess the potential influence of future changes in the larger-scale airflow, particularly changes in NASH extent and intensity, we computed the projected changes between the baseline and future periods in mean monthly 850-hPa geopotential height and airflow, as this pressure level is often used to depict NASH^45^. The projected differences are displayed at 0600 UTC for early (May, Figure S4) and late (August, Fig. 4) in the warm season. The August mean 0600 UTC differences in 850-hPa geopotential heights for the GFDL-driven simulations (RCM3_GFDL, HRM3_GFDL) project the largest increases in geopotential height over the Great Lakes, considerably farther north and west than what might be expected with a strengthening of NASH. However, these projected changes in combination with smaller increases in geopotential height over the Great Plains tighten the geopotential-height gradient over the central plains. The deviations in the vector mean winds also indicate a strengthening of the synoptic-scale airflow over the central plains. In contrast, the CCSM-driven simulations either display little change in the strength of the geopotential-height gradient over the Great Plains (WRFG_CCSM), or a weakening of the gradient and a cyclonic circulation of the vector-wind deviations suggestive of a weaker NASH (CRCM_CCSM). The northerly vector differences in the eastern plains for these two simulations correspond with the locations of projected fewer GPLLJ occurrences seen in Fig. 3. The three CGCM3-driven simulations project larger increases in geopotential height over the northeastern and eastern U.S. compared to the Great Plains. The vector-wind deviations suggest a single center of anticyclonic circulation over New England for WRFG_CGCM3 in contrast to two centers, one over the northeastern U.S. and another over the Gulf of Mexico, for CRCM_CGCM3 and RCM3_CGCM3. The contrasting directions of the difference vectors over the northern Gulf of Mexico and southeastern Texas contribute to the differences between these simulations in projected GPLLJ frequency for the southern plains. The simulated geopotential-height changes for the sole HADCM3-driven simulation contrast sharply with those of the other simulations. There is a moderate height increase over the Gulf of Mexico and bordering states where southerly winds are enhanced, but little change in geopotential height over the rest of the eastern U.S. where wind vector deviations are small and variable. The projected changes in 850 hPa geopotential height and airflow for May (Figure S4) further highlight the similarities between the simulations driven by the same AOGCM, and the differences between the simulations driven by different AOGCMs, in the location of the greatest projected geopotential height increases and enhanced anticyclonic airflow.

**Figure 5:**
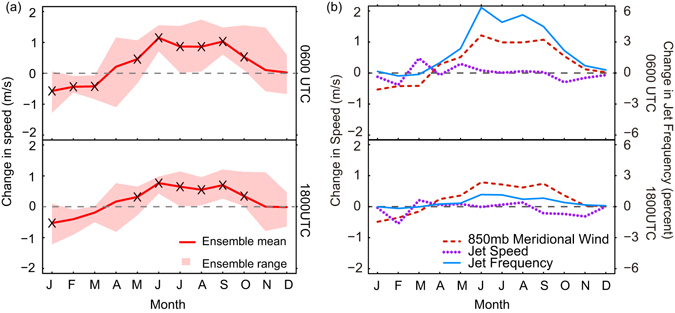
(**a**) Difference of average 850-hPa meridional wind speed for the central plains (see inset in Fig. 1 for location) between the baseline (1970–2000) and mid-century (2040–2070) periods for 0600 UTC (top panel) and 1800 UTC (bottom panel). Gridpoints where the surface pressure was less than 850 hPa were excluded from the calculation of the regional average 850-hPa meridional wind speed. Positive values indicate an increase in mean southerly meridional wind or a decrease in mean northerly meridional wind, and negative values a decrease in mean southerly meridional wind or an increase in mean northerly meridional wind. The solid red line is the multi-model mean difference between the two periods. The red shading depicts the uncertainty range obtained from the eight RCM_AOGCM. The “x” symbols indicate statistically significant differences (p = 0.10) in the multi-model means. Similar plots for additional time steps and for the northern and southern plains are shown in Figure S5. (**b**) Ensemble mean differences between the mid-century (2040–2070) and baseline (1970–2000) periods in 850-hPa meridional wind speed (dashed red line), jet frequency (solid blue line), and jet speed (dotted purple line) for the central plains at 0600 UTC (top panel) and 1800 UTC (bottom panel). Similar plots for additional time steps and for the northern and southern plains are shown in Figure S6. This figure was created in R, version 3.3.1 (R Core Team. (2016). R: A language and environment for statistical computing. R Foundation for Statistical Computing, Vienna, Austria. https://www.R-project.org/).

Lastly, given its previous use as a proxy for the GPLLJ^24, 33^, we assess for each of the three subregions the projected changes in the 850-hPa meridional wind in relation to jet frequency identified above from the vertical wind profiles. Gridpoints where the surface pressure was less than 850 hPa were excluded from the calculation of the regional averages. As illustrated by the 0600 and 1800 UTC time steps for the central region (Fig. 5a, see Figure S5 for additional time steps and regions), only modest seasonal and diurnal fluctuations in the width of the uncertainty range are observed for the projected change in 850-hPa meridional wind speed, in contrast to the large uncertainty ranges observed for the nocturnal 3-hourly periods during the warm season for GPLLJ frequency. The statistically significant differences in the ensemble means suggest a future strengthening of the southerly 850-hPa meridional wind from approximately April through October in the central and southern plains. The differences are largest for 0600 UTC, but the diurnal variations are relatively modest. In the northern region, the mean meridional wind is southerly for only a few summer months, although some strengthening is projected (Figure S5). Only 850-hPa meridional wind speed increases during the nighttime hours are associated with marked increases in jet frequency, particularly for the central plains (Fig. 5b, see Figure S6 for other time steps and regions). Strengthening of the 850-hPa meridional winds at other times of day is only weakly associated with increases in jet frequency. Projected changes in the average speed of GPLLJs that met the selection criteria used in this study are small and display little variation by location and time of day or year in spite of the warm season increase in the broader-scale 850 hPa meridional wind (Fig. 5b). Little change in the relative position of GPLLJ elevation to the elevation of the 850 hPa pressure surface is likely between the baseline and future periods. The projected differences in jet elevation are small, with nocturnal jets located at approximately 500 m AGL for both the baseline and future periods but larger variation among RCM_AOGCM simulations in the baseline and future elevations of GPLLJs occurring in the daytime hours (Figure S7).

## Discussion and Conclusions

Stakeholders benefit from information on potential climate change when it is presented in a format useful for decision making and accompanied with an estimation of the underlying uncertainty. The few earlier studies that considered future changes in the GPLLJ defined the jet in terms of mean meridional airflow on a constant pressure surface. This definition masks the discrete nature of the GPLLJ, as jets occur on some days, or a portion of a day, but not on others^34^. Moreover, future changes presented in terms of differences in mean airflow can be difficult to interpret and incorporate into decision making.

For this study, discrete GPLLJ events were explicitly identified from vertical wind profiles. This approach provides a somewhat different interpretation of the expected changes in the GPLLJ by mid-century compared to those studies that focused on changes in the strength of the meridional wind speed. For instance, projected increases in the 850 hPa meridional wind speed for April-June obtained from a suite of AOGCMs were earlier interpreted as a strengthening of the springtime GPLLJ^24^. A similar increase in the 850 hPa meridional wind speed is projected by the RCM_AOGCM simulations for spring; however, our findings suggest that a strengthening of the broader-scale southerly airflow may lead to more frequent nocturnal jets but not necessarily stronger jets. Moreover, our findings suggest that the largest changes in GPLLJ frequency will occur in summer, in contrast to the small changes in 850 hPa meridional wind obtained earlier from the AOGCM suite^24^. The projected changes in 850 hPa meridional wind speeds obtained when the Weather Research and Forecasting (WRF) model was used to downscale two AOGCMs^33^ also do not align with the projected changes in GPLLJ frequency presented here. The 850 hPa-defined GPLLJ was strongest in April-July and weaker during August-September, whereas the spatial extent of significant changes in jet frequency for the WRFG_CCSM and WRFG_CGCM3 simulations is smaller in May compared to August-September. These contrasting results suggest that multiple, alternative definitions need to be considered when assessing the potential impacts of climate change on the GPLLJ and on complex circulation features in general.

The differences in the projected changes in GPLLJ frequency among the RCM_AOGCM simulations emphasize the importance of multi-model ensembles for robust and flexible decision making. Visual comparison of the spatial patterns of the projected changes from the eight RCM_AOGCM simulations (Figs 2 and 3) suggest that both the choice of driving AOGCM and the RCM have a substantial influence on the interpretation of the future climatology of the GPLLJ. In terms of the driving AOGCM, the simulations forced by CCSM particularly differ from the others, displaying areas of decreased jet frequency in the eastern Great Plains and the Midwest. Another example of the influence of the driving AOGCM is the substantial differences between the HRM3_GFDL and HRM3_HADCM3 projections, with large areas of significant increases in GPLLJ frequency projected when HRM3 is driven by GFDL but little or no change when driven by HADCM3. Biases in the simulated strength of southerly airflow over the Great Plains by the AOGCMs are not necessarily an explanation for the observed differences. For instance, the version of the GFDL AOGCM used for the NARCCAP simulations (GFDL CM2.1, see Table S1) was earlier shown to underestimate, especially in July, the 850 hPa meridional winds when compared to reanalyses (NARR and NCEP)^24^. Yet the projected changes in GPLLJ frequency shown here are larger and more spatially extensive for the GFDL-driven simulations compared to most of the other simulations. In contrast, projected changes in jet frequency during July are relatively small for simulations driven by CGCM3, even though this AOGCM substantially overestimates 850 hPa airflow in the Great Plains at this time of year compared to reanalyses^24^.

Comparisons of the projected changes in GPLLJ frequency obtained from different RCMs but driven by the same AOGCM highlight the influence of the RCMs on our findings. Most notably, the projected changes in GPLLJ frequency are generally smaller for CRCM and WRFG compared to RCM3 and HRM3. This is consistent with the tendency of CRCM and WRFG, when forced by reanalysis fields, to underestimate observed jet frequencies^40^. Comparison of the projections from CRCM_CCSM with those from CRCM_CGCM3 provides further insights on the relative contributions of the RCM. CRCM employed spectral nudging and thus simulations from this RCM should more closely follow the driving AOGCM^46^. Arguably the difference between the CRCM simulations is smaller compared to the differences for the other RCMs, but, nevertheless, the projected future jet frequencies differ substantially between CRCM_CCSM and CRCM_CGCM3, particularly in the latter portion of the warm season. Additional process-based analyses are needed to evaluate the causes of the variations between RCMs, although we suspect that, at least in part, these differences reflect the varying ability of the RCMs to simulate the boundary-layer forcing contributing to GPLLJ occurrence, particularly for nocturnal jets during the warm season. For example, previous authors have suggested that deficiencies in turbulent exchange parameterizations for the stable nocturnal boundary layer may contribute to error in simulating the GPLLJ^26^. GPLLJs during the cool season are more likely to be synoptically forced, which can help explain the greater consensus at this time of year for both the baseline and future periods (Figure S8).

Our analysis also provides insights on the uncertainty surrounding future changes in the large-scale anticyclonic circulation over eastern North America during the warm season. All the NARCCAP simulations indicate a general increase in 850 hPa geopotential height, consistent with thermal expansion in a warmer climate, but the projected changes in the strength and extent of anticyclonic circulation vary among the simulations and do not necessarily agree with earlier studies that attributed future springtime strengthening of low-level meridional airflow in the Great Plains to a westward expansion of NASH^24, 33, 34^ and a weakening of the low-level airflow by late summer to contraction of NASH^33^. Rather, only 50% of the NARCCAP simulations project that NASH will expand westward in May, and only two of the simulations suggest a contraction of NASH in August with the remainder suggesting an expansion of anticyclonic circulation. Moreover, several of the RCM_AOGCM simulations suggest that the greatest geopotential height increases will occur at relatively high latitudes (i.e., over the Great Lakes). Simulating NASH variability and change is a known challenge for AOGCMs^17^, and the differing projections found here suggest this remains a challenge when the AOGCMs are dynamically downscaled.

Climate change assessments primarily have focused on changes in climate parameters such as temperature and precipitation, but projections of future changes in atmospheric circulation features are also essential for adaptation and mitigation planning. The GPLLJ is one of the most significant features of the central U.S., linking the larger-scale circulation with the regional climate. Even modest changes in the GPLLJ will have large impacts. This study is an initial effort to provide projections of future GPLLJ frequency obtained from dynamically-downscaled climate model simulations and to convey the uncertainty associated with the projections. The considerable inter-model variability in the projected changes of the future GPLLJ climatology supports the recommendations of previous authors that a suite of downscaled climate simulations obtained from multiple RCMs and AOGCM is essential for exploring the uncertainty of future projections^46^. This uncertainty must be considered by stakeholders to help make decisions that are robust to a range of future changes.

### Data Availability

The North American Regional Climate Change Assessment Program (NARCCAP) dynamically-downscaled projections that were analyzed in this study are available at http://www.narccap.ucar.edu/. Datasets generated during the current study are available from the corresponding author on reasonable request.

## Electronic supplementary material

Supplementary Information
