# Supplementary material for: Future changes in the climatology of the Great Plains low-level jet derived from fine resolution multi-model simulations
Source: Sci Rep. 2017 Jul 10;7:5029. doi: 10.1038/s41598-017-05135-0 (PMC5504071; doi:10.1038/s41598-017-05135-0)
Supplement: Supplementary file 1 — Supplementary Information [file 41598_2017_5135_MOESM1_ESM.doc]

Supplementary Information

**Future changes in the climatology of the Great Plains low-level jet derived from fine resolution multi-model simulations**

Ying Tang^a^, Julie Winkler^a^, Shiyuan Zhong^a^, Xindi Bian^b^, Dana Doubler^a^, Lejiang Yu^a^, and Claudia Walters^c^

^a^Department of Geography, Environment and Spatial Sciences, Michigan State University, East Lansing, Michigan, 48824

^b^Northern Research Station, USDA Forest Service, Lansing, Michigan, 48910

^c^Department of Social Science, University of Michigan-Dearborn, Dearborn, Michigan, 48128

**Significance Testing:** Several approaches were used to test for significant differences in the multi-model means of the projected changes in the frequency of the Great Plains low-level jet (GPLLJ) between the baseline and future periods. As a first step, we simply summarized the agreement of the sign of the projected change, as shown in the top row of plots in Figure S2 below for the three spatial windows (northern plains, central plains, southern plains). For the significance testing reported in the main text, we assumed that the eight RCM_AOGCM combinations represent a sample of all possible dynamically-downscaled projections under the SRES A2 emissions scenario. For each of the three spatial windows, the monthly jet frequencies were averaged by year across the RCM_AOGCM combinations for the baseline and mid-century periods, and the differences in the multi-model means between the two periods were tested for statistical significance using Student’s t-test with unequal variance applied to the 30-year arrays of multi-model means (degrees of freedomare calculated in R using the [Welch–Satterthwaite equation](https://en.wikipedia.org/wiki/Welch–Satterthwaite_equation)). The significance values by month and 3-hourly time step are shown in the second row of plots in Figure S2, although they should be interpreted cautiously as the RCM_AOGCM simulations may not be independent. In addition, we tested the 30-year average values (one mean for each model for the control and future time periods) for significance using Student’s t-test assuming unequal variance (degrees of freedom calculated in R using [Welch–Satterthwaite equation](https://en.wikipedia.org/wiki/Welch–Satterthwaite_equation)). This is a stricter test given the smaller number of degrees of freedom, and significant differences were found only for nocturnal jet frequencies in the central plains. Finally, we focused explicitly on the eight RCM_AOGCM combinations, rather than considering them a sample of a larger population of dynamically-downscaled projections, and performed a paired t-test (degrees of freedom equal 7). The significance levels for the paired t-test are shown in the third row of Figure S2.

**Table S1.** The RCM_AOGCM simulation used in the current study and abbreviations for model names.

| **RCM_AOGCM combination** | **RCM** | **AOGCM** |
| --- | --- | --- |
| **CRCM_CCSM** | Canadian Regional Climate Model (CRCM) | NCAR Community Climate Model (CCSM) version 3 |
| **CRCM_CGCM3** | Canadian Regional Climate Model (CRCM) | Canadian Global Climate Model version 3 (CGCM3) |
| **WRFG_CCSM** | Weather Research & Forecasting model with Grell-Devenyi Cumulus Scheme (WRFG) | NCAR Community Climate Model (CCSM) version 3 |
| **WRFG_CGCM3** | Weather Research & Forecasting model with Grell-Devenyi Cumulus Scheme (WRFG) | Canadian Global Climate Model version 3 (CGCM3) |
| **RCM3_GFDL** | Regional Climate Model version 3 (RCM3) | Geophysical Fluid Dynamics Laboratory (GFDL) Climate Model version 2.1 (CM2.1) |
| **RCM3_CGCM3** | Regional Climate Model version 3 (RCM3) | Canadian Global Climate Model version 3 (CGCM3) |
| **HRM3_GFDL** | Hadley Regional Model (HRM3) | Geophysical Fluid Dynamics Laboratory (GFDL) Climate Model version 2.1 (CM2.1) |
| **HRM3_HADCM3** | Hadley Regional Model 3 (HRM3) | United Kingdom (UK) Hadley Centre Climate Model version 3 (HADCM3) |

**
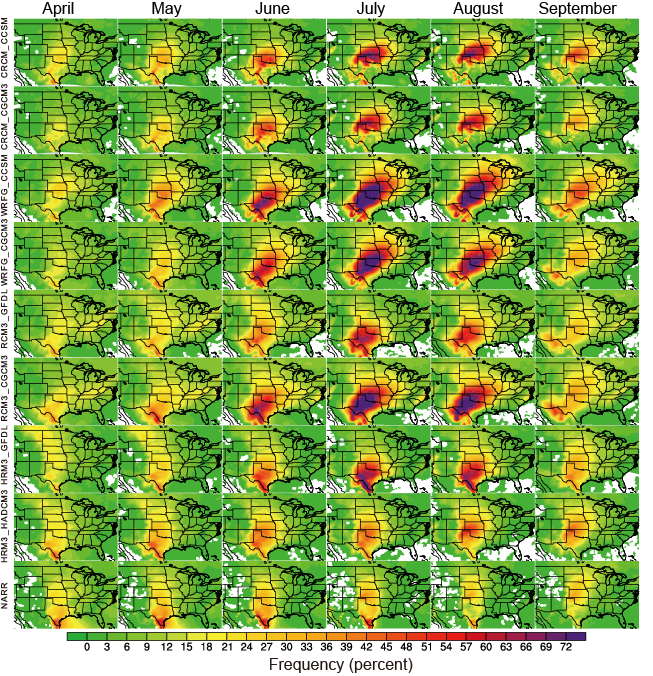
**

**Figure S1.** Frequency of Great Plains low-level jets (GPLLJs) from April to September at 0600 UTC for eight dynamically-downscaled climate simulations obtained from the North American Regional Climate Change Assessment Program (NARCCAP) for a baseline period (1970-2000), and, in the bottom row, for a historical (1979-2009) period obtained from the North American Regional Reanalysis (NARR). The frequencies are expressed as a percentage of the total number of time steps during the respective summary periods. The NARR frequencies are remapped from Doubler et al. (2015) (citation #21 in main text). The NARCCAP simulations are labeled by the regional climate model (RCM) and driving atmospheric ocean general circulation model (AOGCM). This figure was created using NCAR Command Language (NCL) Version 6.3.0 (The NCAR Command Language (Version 6.3.0) [Software]. (2016). Boulder, Colorado: UCAR/NCAR/CISL/TDD. http://dx.doi.org/10.5065/D6WD3XH5).

**
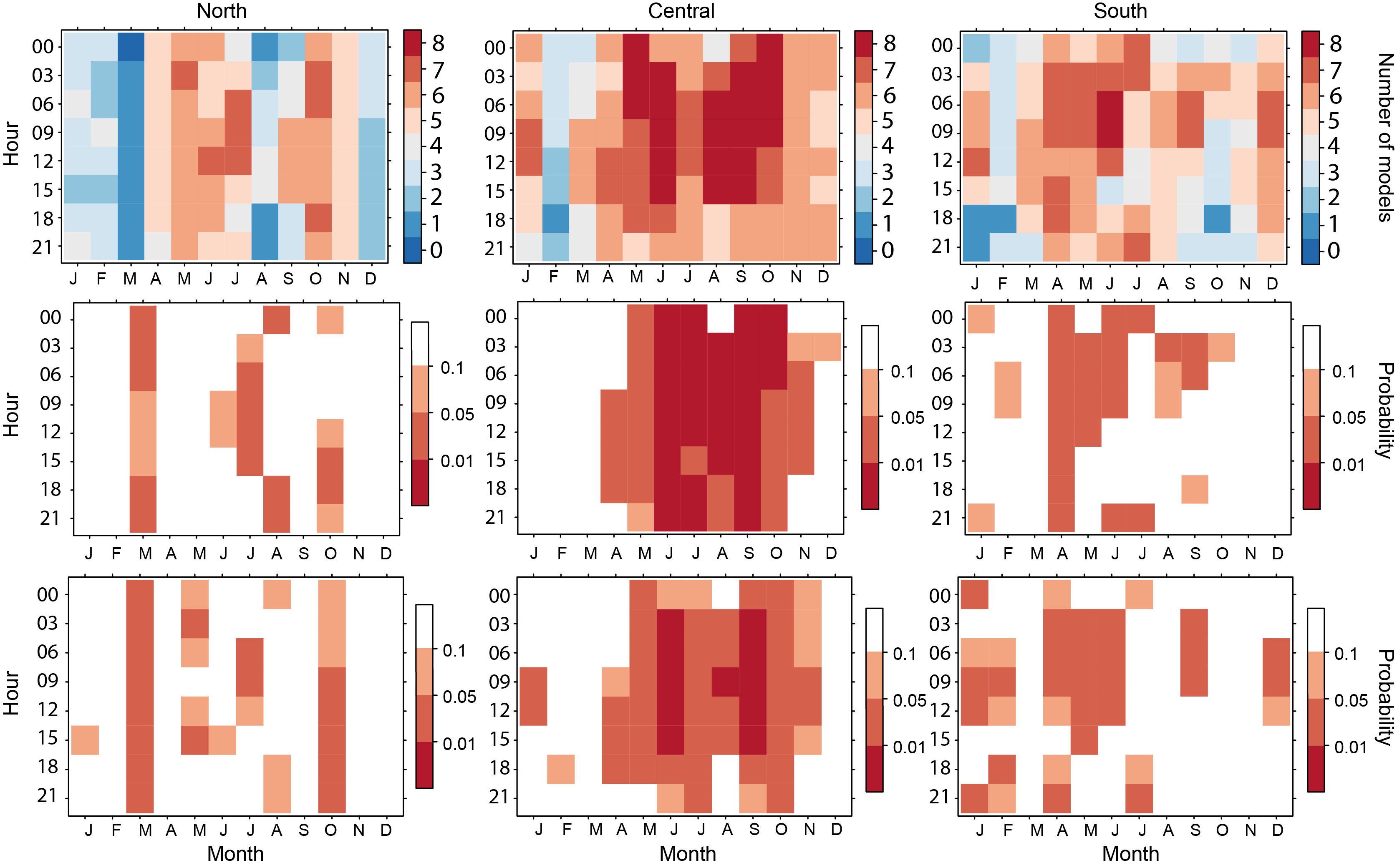
**

Figure S2. Significance testing by 3-hourly time step and month of the projected changes in the frequency of the Great Plains low-level jet (GPLLJ) between baseline and future periods for the three spatial averaging windows (northern plains, central plains, and southern plains). The top row displays the agreement in the sign of the projected changes, with the brown tones indicating that the majority of the models projected an increase in jet frequency and the blue tones indicating that the majority of the models projected a decrease in jet frequency. The middle row shows the significance level for the significance testing reported in the main text using Student’s t-test with unequal variance. The Student’s t-test was performed on the 30-year arrays of multi-model means (degrees of freedom was calculated in R using the [Welch–Satterthwaite equation](https://en.wikipedia.org/wiki/Welch–Satterthwaite_equation)) obtained from the monthly jet frequencies averaged by year across the RCM_AOGCM combinations for the baseline and mid-century periods. The last row shows the significance level of a paired t-test on the 30-year average monthly jet frequency from eight RCM_AOGCM combinations (degrees of freedom = 7) for the baseline and mid-century periods. The colors in the plots for the middle and last row, ranging from dark to light, refer to p ≤ .01, p ≤ .05, and p ≤ .10. This figure was created in R, version 3.3.1 (R Core Team. (2016). R: A language and environment for statistical computing. R Foundation for Statistical Computing, Vienna, Austria. URL https://www.R-project.org/).


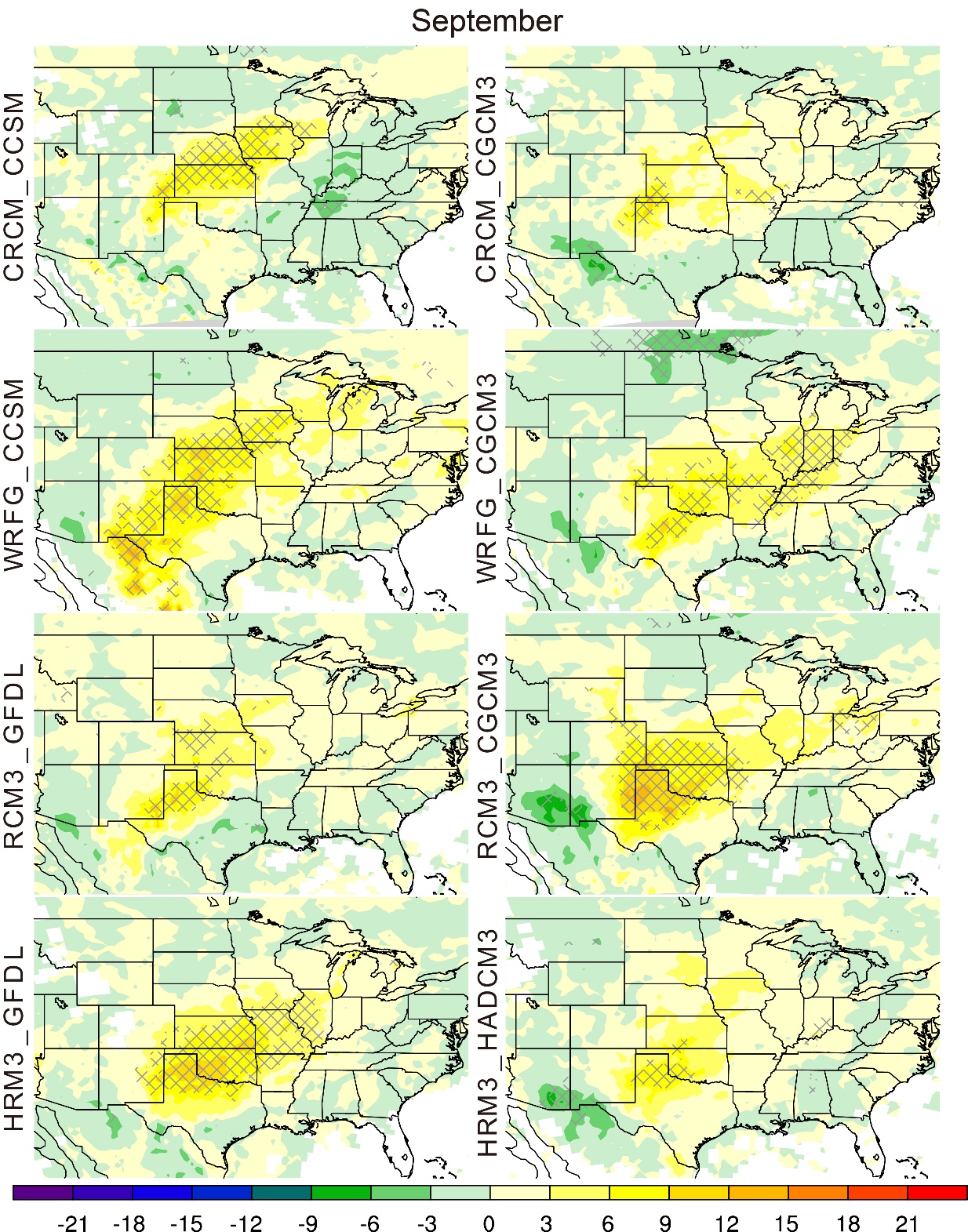


**Figure S3.** Projected change by mid-century in the frequency (in percent) of GPLLJs at 0600 UTC during September for each of the 8 RCM_AOGCM simulations. Hatching indicates gridpoints with statistically significant differences (p=0.10) between the mid-century and baseline periods. This figure was created using NCAR Command Language (NCL) Version 6.3.0 (The NCAR Command Language (Version 6.3.0) [Software]. (2016). Boulder, Colorado: UCAR/NCAR/CISL/TDD. http://dx.doi.org/10.5065/D6WD3XH5).


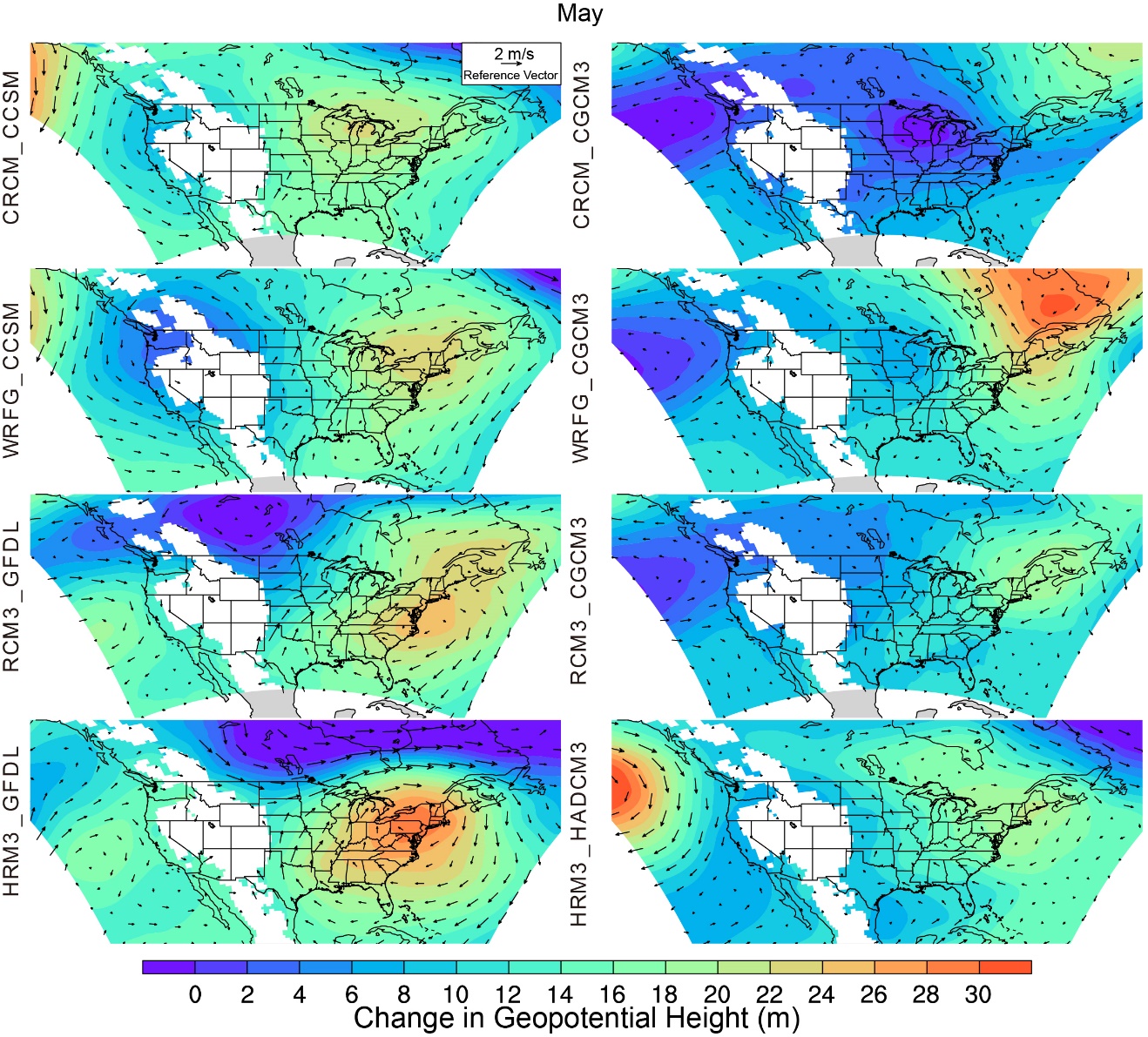


**Figure S4.** Differences in 850-hPa geopotential height (shading) and 850-hPa wind (vectors) between the mid-century and baseline periods at 0600 UTC for May for each of the 8 RCM_AOGCM simulations. Gridpoints with elevations greater than 1400 m above sea level are masked in white. This figure was created using NCAR Command Language (NCL) Version 6.3.0 (The NCAR Command Language (Version 6.3.0) [Software]. (2016). Boulder, Colorado: UCAR/NCAR/CISL/TDD. http://dx.doi.org/10.5065/D6WD3XH5).

**
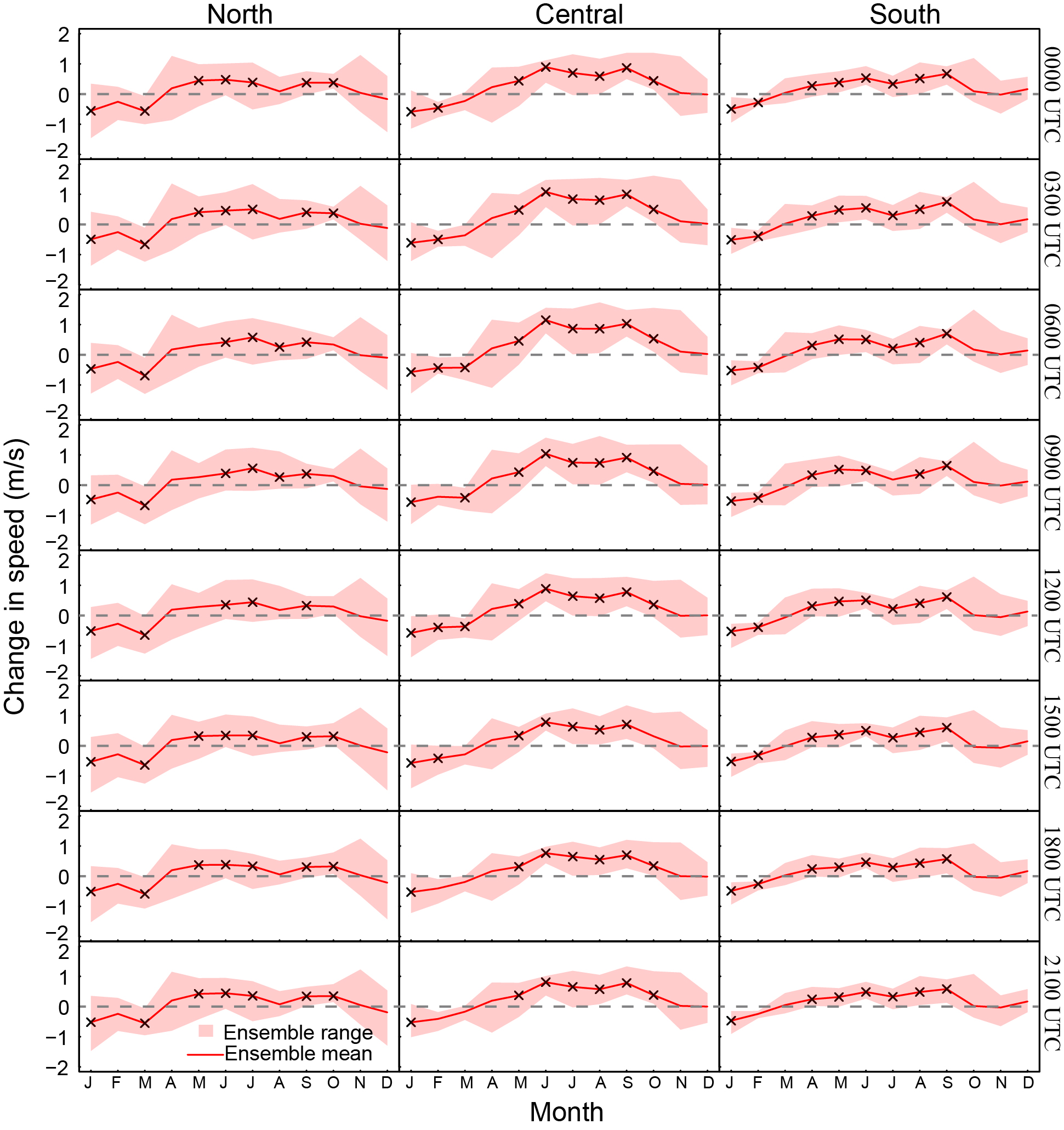
**

**Figure S5.** The difference of average 850-hPa meridional wind speed for each of the 3-hour time periods for the 3 subregions shown in Figure 1 between the baseline (1970-2000) and mid-century (2040-2070) periods. Gridpoints within the 3 regions where the surface pressure was less than 850 hPa were excluded from the calculation of the regional average 850-hPa meridional wind speed. Positive values indicate a mean southerly meridional wind, and negative values a mean northerly meridional wind. The solid red line is the multi-model mean, and the red shading depicts the uncertainty range obtained from the 8 RCM-AOGCM simulations. Symbols (“x”) indicate statistically significant differences (p=0.10) in the multi-model means. The dashed black line represents the zero line. This figure was created in R, version 3.3.1 (R Core Team. (2016). R: A language and environment for statistical computing. R Foundation for Statistical Computing, Vienna, Austria. URL https://www.R-project.org/).


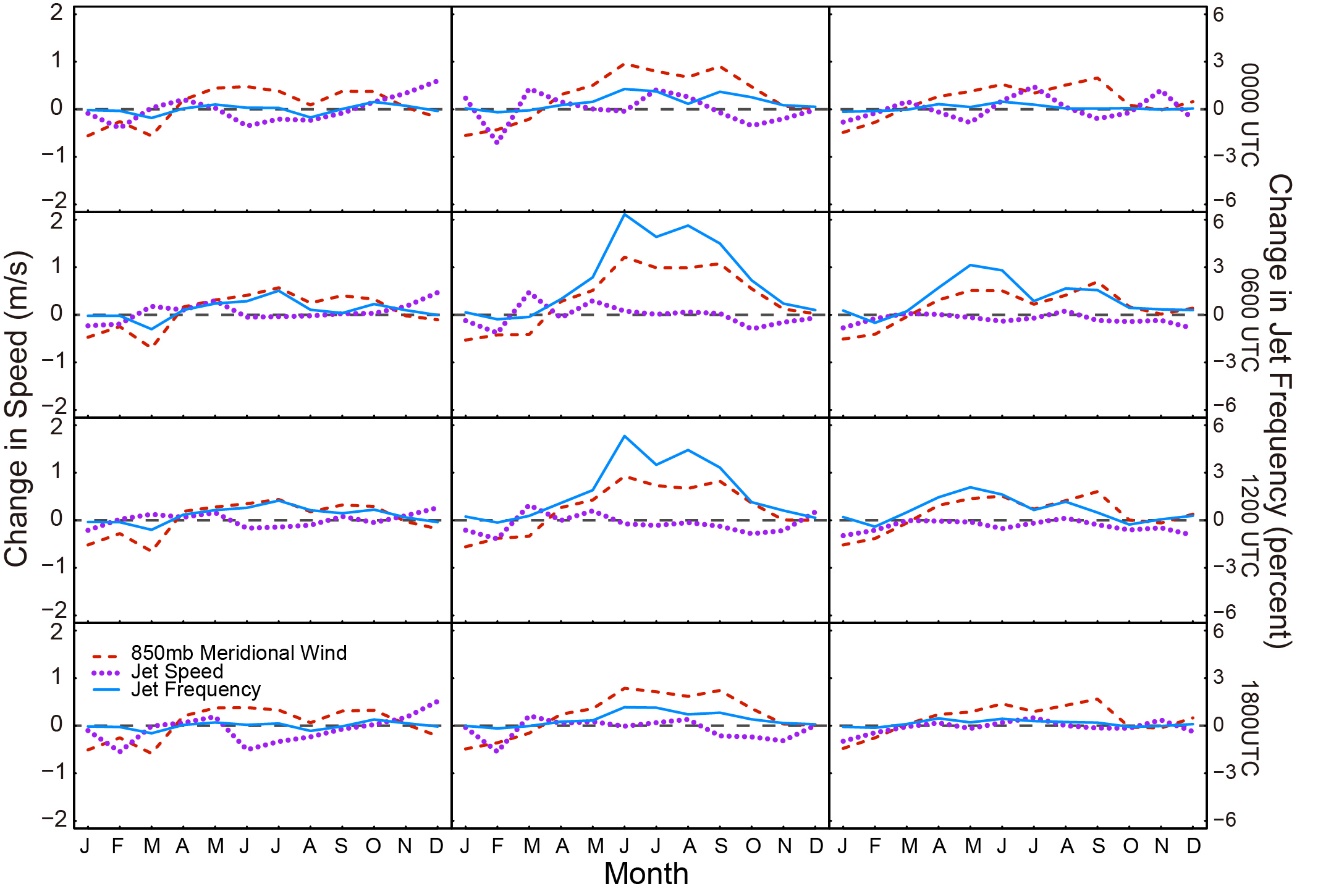


**Figure S6.** Ensemble mean differences by 6-hourly time steps between the mid-century and baseline periods for 850-hPa meridional wind speed (dashed red line), jet frequency (solid blue line), and jet speed (dashed purple line) for the 3 regions shown in Figure 1. The dashed black line represents the zero line. This figure was created in R, version 3.3.1 (R Core Team. (2016). R: A language and environment for statistical computing. R Foundation for Statistical Computing, Vienna, Austria. URL https://www.R-project.org/).

**
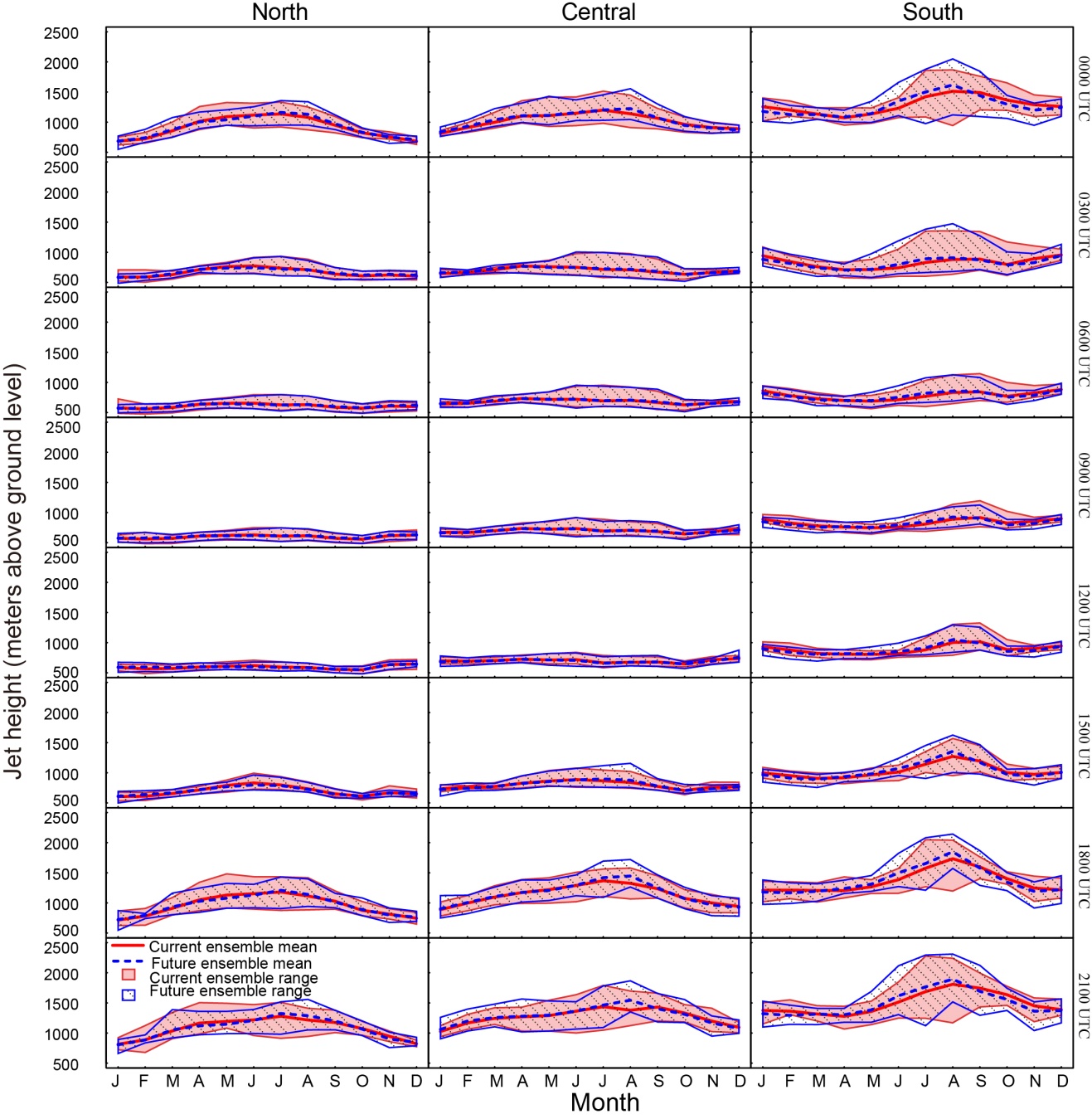
**

**Figure S7.** Average height for GPLLJ for each of the 3-hour time periods for the 3 subregions shown in Figure 1 between the baseline (1970-2000) and mid-century (2040-2070) periods. The solid red line is the multi-model mean for the baseline period, and the dashed blue line is the multi-model mean for the mid-century period. The red shading depicts the uncertainty range obtained from the 8 RCM-AOGCM simulations for the baseline period, and the solid blue lines and hatching depict the uncertainty range for the mid-century period. This figure was created in R, version 3.3.1 (R Core Team. (2016). R: A language and environment for statistical computing. R Foundation for Statistical Computing, Vienna, Austria. URL https://www.R-project.org/).

**
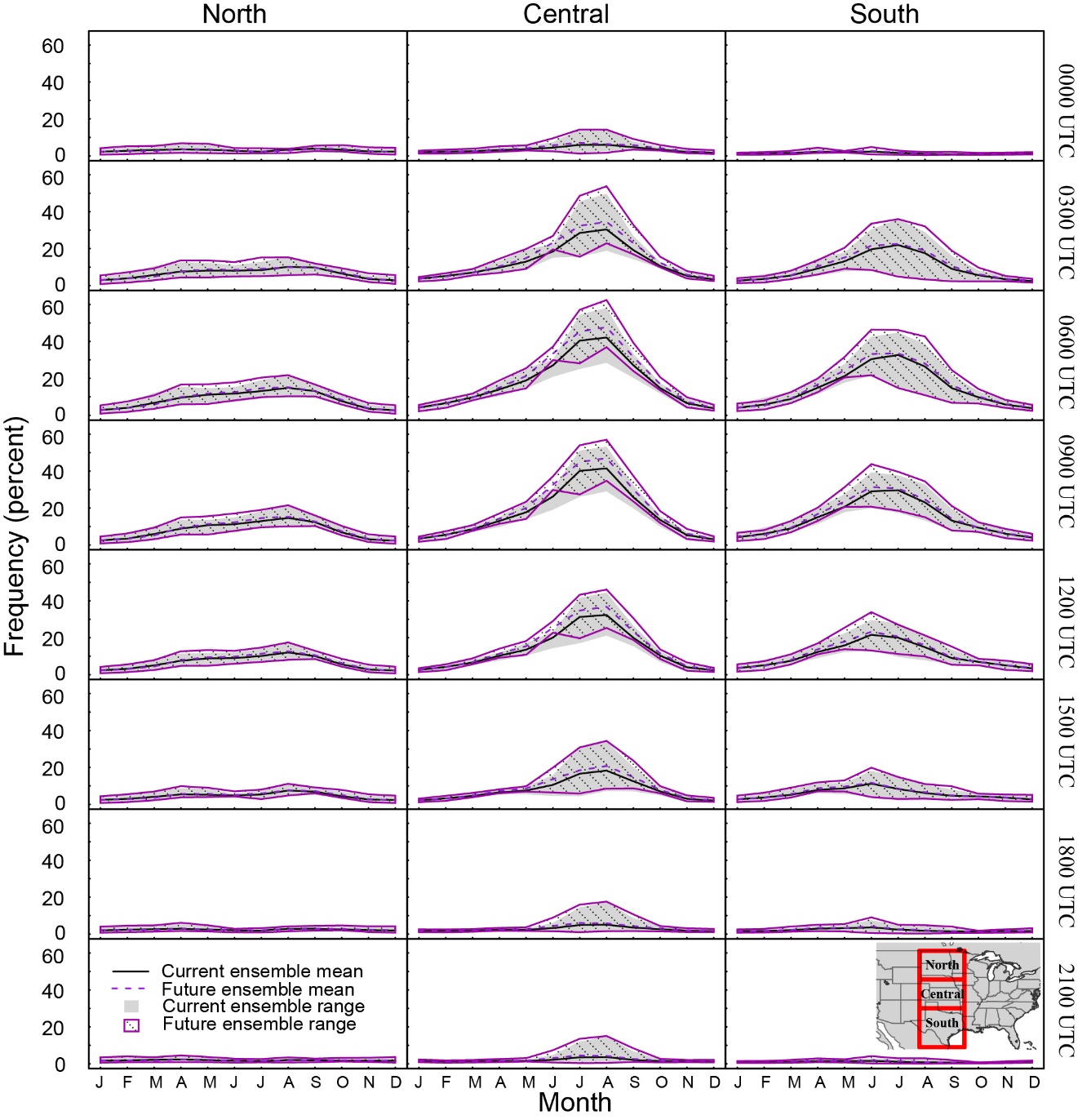
**

**Figure S8.** Average GPLLJ frequency for 3 subregions over the Great Plains by month for the baseline (1970-2000) and mid-century (2040-2070) periods. Monthly frequencies are shown for each of the 3-hour time periods available for the NARCCAP simulations. The locations of the subregions are shown in the inset map. The solid black line is the multi-model mean for the baseline period, and the dashed purple line is the multi-model mean for the mid-century period. The gray shading depicts the uncertainty range obtained from the 8 RCM_AOGCM simulations for the baseline period and the solid purple lines and hatching depict the uncertainty range for the mid-century period. The symbols indicate statistically significant differences (p=0.10) in the multi-model means. This figure was created in R, version 3.3.1 (R Core Team. (2016). R: A language and environment for statistical computing. R Foundation for Statistical Computing, Vienna, Austria. URL https://www.R-project.org/).
